# NOMINATA

**Published:** 2017

We thank all reviewers who, during 2017, helped to produce the Revista Brasileira de Terapia Intensiva with dedication and enthusiasm.

**Table:** 

Abel Arroyo-Sanchez	Hospital Victor Lazarte Echegaray	Trujillo, Peru
Adriana Kessler	Universidade Federal de Ciências da Saúde de Porto Alegre	Porto Alegre (RS), Brazil
Alexandre Carvalho	UDI Hospital	São Luis (MA), Brazil
Ana Cristina Tannuri	Faculdade de Medicina, Universidade de São Paulo	São Paulo (SP), Brazil
Ana Luiza Mezzaroba	Universidade Estadual de Londrina	Londrina (PR), Brazil
Ana Maria Wilson	Escola de Enfermagem, Universidade de São Paulo	São Paulo (SP), Brazil
André Gustavo de Albuquerque	Instituto Nacional de Cancer	Rio de Janeiro (RJ), Brazil
André Kalil	Medical Center, University of Nebraska	Nebraska, United States
André Miguel Japiassú	Instituto de Pesquisa Clínica Evandro Chagas	Rio de Janeiro (RJ), Brazil
Andres Aquevedo	Pontificia Universidad Catolica de Chile	Santiago, Chile
Anelise Fonseca	Hospital Adventista Silvestre	Rio de Janeiro (RJ), Brazil
Angela Ferreira	Hospital do Coração	São Paulo (SP), Brazil
Ângelo Miranda Rocha	CESMAC	Maceió (AL), Brazil
Antônio Paulo Nassar Júnior	Hospital das Clínicas, Faculdade de Medicina, Universidade de São Paulo	São Paulo (SP), Brazil
Arnaldo Dubin	Sanatorio Otamendi y Miroli	Buenos Aires, Argentina
Arnaldo Prata Barbosa	Universidade Federal do Rio de Janeiro	Rio de Janeiro (RJ), Brazil
Artur Delgado	Instituto da Criança, Hospital das Clínicas, Faculdade de Medicina, Universidade de São Paulo	São Paulo (SP), Brazil
Arturo Briva	Hospital de Clínicas	Montevideo, Uruguay
Ary Serpa Neto	Hospital Israelita Albert Einstein	São Paulo (SP), Brazil
Bruno Besen	Hospital das Clínicas, Faculdade de Medicina, Universidade de São Paulo	São Paulo (SP), Brazil
Bruno Tomazini	Instituto de Ensino e Pesquisa, Hospital Sírio-Libanês	São Paulo (SP), Brazil
Camila Dietrich	Universidade Federal de Ciências da Saúde de Porto Alegre	Porto Alegre (RS), Brazil
Carla Nicolau	Instituto da Criança, Hospital das Clínicas, Faculdade de Medicina, Universidade de São Paulo	São Paulo (SP), Brazil
Carlos Toufen Júnior	Instituto da Criança, Hospital das Clínicas, Faculdade de Medicina, Universidade de São Paulo	São Paulo (SP), Brazil
Carmem Sílvia Valente Barbas	Faculdade de Medicina, Universidade de São Paulo	São Paulo (SP), Brazil
Carolina Amoretti	Hospital São Rafael	Salvador (BA), Brazil
Carolina Fu	Faculdade de Medicina, Universidade de São Paulo	São Paulo (SP), Brazil
Cássia Righy Shinotsuka	Hospital Copa D'Or	Rio de Janeiro (RJ), Brazil
Cássia Vancini-Campanharo	Universidade Federal de São Paulo	São Paulo (SP), Brazil
Catarina Conceição	Hospital São Francisco Xavier, Centro Hospitalar de Lisboa Central	Lisbon, Portugal
Cecília Couto	Universidade Federal do Rio Grande do Sul	Porto Alegre (RS), Brazil
Cecília Draque	Escola Paulista de Medicina, Universidade Federal de São Paulo	São Paulo (SP), Brazil
Cíntia Grion	Universidade Estadual de Londrina	Londrina (PR), Brazil
Cléa Rodrigues Leone	Instituto da Criança, Hospital das Clínicas, Faculdade de Medicina, Universidade de São Paulo	São Paulo (SP), Brazil
Corinne Taniguchi	Hospital Israelita Alberto Einstein	São Paulo (SP), Brazil
Cristian Tonial	Pontifícia Universidade Católica do Rio Grande do Sul	Porto Alegre (RS), Brazil
Cristiane Tomasi	Universidade do Extremo Sul Catarinense	Criciúma (SC), Brazil
Cristina Granja	Centro Hospitalar do Algarve	Faro, Portugal
Daniel Neves Forte	Hospital Sírio-Libanês	São Paulo (SP), Brazil
Daniela Fiorenzano	Instituto da Criança, Hospital das Clínicas, Faculdade de Medicina, Universidade de São Paulo	São Paulo (SP), Brazil
Daniela Souza	Hospital Universitário, Universidade de São Paulo	São Paulo (SP), Brazil
David Nora	Hospital de Vila Franca de Xira	Vila Franca de Xira, Portugal
Dimitri Gusmao-Flores	Instituto de Ciência da Saúde, Universidade Federal da Bahia	Salvador (BA), Brazil
Edison Rodrigues Filho	Hospital Dom Vicente Scherer - Santa Casa de Misericórdia de Porto Alegre	Porto Alegre (RS), Brazil
Edna Maria Diniz	Instituto da Criança, Hospital das Clínicas, Faculdade de Medicina, Universidade de São Paulo	São Paulo (SP), Brazil
Eduardo Costa	Faculdade de Medicina, Universidade de São Paulo	São Paulo (SP), Brazil
Eduardo Mekitarian Filho	Universidade de São Paulo	São Paulo (SP), Brazil
Eduardo Osawa	Faculdade de Medicina, Universidade de São Paulo	São Paulo (SP), Brazil
Elidio Angioletto	Universidade do Extremo Sul Catarinense	Criciúma (SC), Brazil
Elisa Estenssoro	Hospital Interzonal de Agudos San Martin de La Plata	Buenos Aires, Argentina
Ellen Gaetti-Jardim	Universidade Federal do Mato Grosso do Sul	Campo Grande (MS), Brazil
Fabiane Backes	Hospital de Clínicas de Porto Alegre	Porto Alegre (RS), Brazil
Fabiano Nagel	Hospital de Clínicas de Porto Alegre	Porto Alegre (RS), Brazil
Felipe Saddy	Hospital Copa D'Or	Rio de Janeiro (RJ), Brazil
Fernanda Kutchak	Universidade Vales dos Sinos	Porto Alegre (RS), Brazil
Fernando Godinho Zampieri	Hospital das Clínicas, Faculdade de Medicina, Universidade de São Paulo	São Paulo (SP), Brazil
Fernando Rios	Hospital Nacional Profesor Alejandro Posadas	Buenos Aires, Argentina
Fernando Spiller	Universidade Federal de Santa Catarina	Florianópolis (SC), Brazil
Fernando Suparregui Dias	Hospital Pompeia	Caxias do Sul (RS), Brazil
Flávia Ribeiro Machado	Universidade Federal de São Paulo	São Paulo (SP), Brazil
Flávio Freitas	Universidade Federal de São Paulo	São Paulo (SP), Brazil
Francisco Bruno	Hospital São Lucas, Pontifícia Universidade Católica do Rio Grande do Sul	Porto Alegre (RS), Brazil
Francisco Garcia Soriano	Faculdade de Medicina, Universidade de São Paulo	São Paulo (SP), Brazil
Francisco Hurtado	Faculdad de Medicina, Universidad de La Republica	Montevideo, Uruguay
Frederico Bruzzi de Carvalho	Fundação Hospitalar do Estado de Minas Gerais	Belo Horizonte (NG), Brazil
Gilberto Friedman	Universidade Federal do Rio Grande do Sul	Porto Alegre (RS), Brazil
Giuliana Moralez	Hospital Estadual Getúlio Vargas	Rio de Janeiro (RJ), Brazil
Glauco Westphal	Hospital Municipal São José	Joinville (SC), Brazil
Gustavo Adolfo Ospina-Tascón	Fundación Valle del Lili	Valle del Cauca, Colombia
Heitor Pons Leite	Universidade Federal de São Paulo	São Paulo (SP), Brazil
Hélio Penna Guimarães	Hospital do Coração	São Paulo (SP), Brazil
Hélio Queiroz	Hospital da Criança, Obras Sociais Irmã Dulce	Salvador (BA), Brazil
Ignacio Martin-Loeches	St. James University Hospital, Trinity Centre for Health Sciences	Dublin, Ireland
Irene Aragão	Centro Hospitalar do Porto	Porto, Portugal
Isabela Freire Azevedo Santos	Universidade Federal do Sergipe	São Cristóvão (SE), Brazil
Israel Maia	Hospital Nereu Ramos	Florianópolis (SC), Brazil
Ivens Souza	Hospital Sírio-Libanês	São Paulo (SP), Brazil
Jan Bakker	Erasmus University Medical Center	Rotterdam, Netherlands
Joana Silvestre	Universidade Nova de Lisboa	Lisbon, Portugal
João Gabriel Ramos	Hospital São Rafael	Salvador (BA), Brazil
João Gonçalves Pereira	Hospital de Vila Franca de Xira	Vila Franca de Xira, Portugal
João Manoel Silva Júnior	Hospital do Servidor Público Estadual	São Paulo (SP), Brazil
João Wilney	InGastro	Porto Alegre (RS), Brazil
José A Lorente	Hospital Universitário de Getafe	Madrid, Spain
José Augusto da Silva	Fundação de Beneficência Hospital de Cirurgia	Aracaju (SE), Brazil
José Colleti Júnior	Hospital Santa Catarina	São Paulo (SP), Brazil
José Roberto Fioretto	Faculdade de Medicina de Botucatu, Universidade Estadual Paulista	Botucatu (SP), Brazil
José Rodolfo Rocco	Hospital Universitário Clementino Fraga Filho, Universidade Federal do Rio de Janeiro	Rio de Janeiro (RJ), Brazil
Juang Horng Jyh	Hospital Municipal "Dr. Carmino Caricchio"	São Paulo (SP), Brazil
Larissa Constantino	Universidade do Extremo Sul Catarinense	Criciúma (SC), Brazil
Laura dos Santos	Universidade Luterana do Brazil	Canoas (RS), Brazil
Lígia Rabello	Instituto Nacional de Câncer	Rio de Janeiro (RJ), Brazil
Lília Nogueira	Escola de Enfermagem, Universidade de São Paulo	São Paulo (SP), Brazil
Lilian Tanaka	Hospital Copa D'Or	Rio de Janeiro (RJ), Brazil
Livia Andrade	Instituto de Medicina Integral Professor Fernando Figueira	Recife (PE), Brazil
Livia Barbosa	Hospital Sírio-Libanês	São Paulo (SP), Brazil
Lívia Melro	Hospital das Clínicas, Faculdade de Medicina, Universidade de São Paulo	São Paulo (SP), Brazil
Lúcia Borges	Universidade Federal de Ciências da Saúde de Porto Alegre	Porto Alegre (RS), Brazil
Luciana Chiavegato	Universidade Cidade de São Paulo	São Paulo (SP), Brazil
Luciano César Pontes de Azevedo	Universidade de São Paulo	São Paulo (SP), Brazil
Luis Bento	Centro Hospilar Lisboa Central, EPE	Lisbon, Portugal
Luis Coelho	Hospital São Francisco Xavier, Centro Hospitalar de Lisboa Central	Lisbon, Portugal
Luiz Malbouisson	Hospital das Clínicas, Faculdade de Medicina, Universidade de São Paulo	São Paulo (SP), Brazil
Michael Czaplik	University Hospital Aachen, RWTH Aachen University	Aachen, Germany
Manuel Jibaia	Hospital Eugenio Espejo	Quito, Ecuador
Marcella Almeida	Universidade de São Paulo	São Paulo (SP), Brazil
Marcelo Beraldo	Universidade de São Paulo	São Paulo (SP), Brazil
Marcelo Brandão	Universidade Estadual de Campinas	Campinas (SP), Brazil
Marcelo Park	Hospital das Clínicas, Faculdade de Medicina, Universidade de São Paulo	São Paulo (SP), Brazil
Márcia Morete	Hospital Israelita Albert Einstein	São Paulo (SP), Brazil
Márcio Osório Guerreiro	Hospital São Francisco de Paula	Pelotas (RS), Brazil
Márcio Manozzo Boniatti	Hospital de Clínicas de Porto Alegre	Porto Alegre (RS), Brazil
Márcio Soares	Instituto D'Or de Pesquisa e Ensino	Rio de Janeiro (RJ), Brazil
Marcos Paula	Universidade do Sul de Santa Catarina	Tubarão (SC), Brazil
Marcus Andrade	Faculdade de Medicina, Universidade Federal de Minas Gerais	Belo Horizonte (MG), Brazil
Maria Augusta Gibelli	Instituto da Criança, Hospital das Clínicas, Faculdade de Medicina, Universidade de São Paulo	São Paulo (SP), Brazil
Maria Carolina Paulino	Hospital São Francisco Xavier	Lisbon, Portugal
Maria Esther Ceccon	Instituto da Criança, Hospital das Clínicas, Faculdade de Medicina, Universidade de São Paulo	São Paulo (SP), Brazil
Maria Helena Dittrich	Hospital Infantil Sabará	São Paulo (SP), Brazil
Marilyn Ferreira	Associação Educacional Luterana Bom Jesus/IELUSC	Joinville (SC), Brazil
Marta M Mataloun	Instituto da Criança, Hospital das Clínicas, Faculdade de Medicina, Universidade de São Paulo	São Paulo (SP), Brazil
Mavilde Pedreira	Universidade Federal de São Paulo	São Paulo (SP), Brazil
Monique Michels	Universidade do Extremo Sul Catarinense	Criciúma (SC), Brazil
Nelson Akamine	Associação Paulista para o Desenvolvimento da Medicina	São Paulo (SP), Brazil
Nelson Horigoshi	Hospital Infantil Sabará	São Paulo (SP), Brazil
Nicolas Nin	Hospital de Torrejon	Madrid, Spain
Norma Suely	Universidade Federal do Espírito Santo	Vitória (ES), Brazil
Nuno Carvalho	Hospital Garcia de Orta, EPE	Setúbal, Portugal
Oellen Franzosi	Universidade Federal do Rio Grande do Sul	Porto Alegre (RS), Brazil
Otávio Ranzani	Hospital das Clínicas, Faculdade de Medicina, Universidade de São Paulo	São Paulo (SP), Brazil
Patrícia Zamberlan	Instituto da Criança, Hospital das Clínicas, Faculdade de Medicina, Universidade de São Paulo	São Paulo (SP), Brazil
Paulo Martins	Centro Hospitalar e Universitário de Coimbra	Coimbra, Portugal
Pedro Branco	Hospital São José	Lisbon, Portugal
Pedro Celiny Ramos Garcia	Pontifícia Universidade Católica do Rio Grande do Sul	Porto Alegre (RS), Brazil
Pedro Kurtz	Instituto Estadual do Cérebro Paulo Niemeyer	Rio de Janeiro (RJ), Brazil
Pedro Mendes	Hospital das Clínicas, Faculdade de Medicina, Universidade de São Paulo	São Paulo (SP), Brazil
Pedro Póvoa	Hospital São Francisco Xavier, Centro Hospitalar de Lisboa Central	Lisbon, Portugal
Péricles Duarte	Unioeste	Cascavel (PR), Brazil
Ramon Costa	AC Camargo Cancer Center	São Paulo (SP), Brazil
Raphael Augusto	Universidade de São Paulo	São Paulo (SP), Brazil
Raquel Annoni	Universidade Federal do Triângulo Mineiro	Uberaba (MG), Brazil
Régis Goulart Rosa	Hospital Moinhos de Vento	Porto Alegre (RS), Brazil
Renata Teixeira Ladeira	Instituto Ladeira & Barros	Barra do Bugres (MT), Brazil
Renata Yoshida	Faculdade de Medicina, Universidade de São Paulo	São Paulo (SP), Brazil
Ricardo Castro	Universidade de Pittsburgh	Pittsburgh, United States
Ricardo Cordioli	Hospital Israelita Albert Einstein	São Paulo (SP), Brazil
Ricardo Kenji Nawa	Hospital Sírio-Libanês	São Paulo (SP), Brazil
Ricardo Yamaguchi	Assistência Médica Internacional SA	São Paulo (SP), Brazil
Rita Silveira	Universidade Federal do Rio Grande do Sul	Porto Alegre (RS), Brazil
Robson Amorim	Hospital das Clínicas, Faculdade de Medicina, Universidade de São Paulo	São Paulo (SP), Brazil
Rodrigo Ghedini	Hospital de Clínicas de Porto Alegre	Porto Alegre (RS), Brazil
Rodrigo Serafim	Universidade Federal do Rio de Janeiro	Rio de Janeiro (RJ), Brazil
Rogério Zigaib	Hospital Paulistano	São Paulo (SP), Brazil
Romy Brock	Instituto da Criança, Hospital das Clínicas, Faculdade de Medicina, Universidade de São Paulo	São Paulo (SP), Brazil
Roselaine de Oliveira	Hospital Moinhos de Vento	Porto Alegre (RS), Brazil
Rosemeri Maurici	Universidade Federal de Santa Catarina	Florianópolis (SC), Brazil
Rubens Feferbaum	Instituto da Criança, Hospital das Clínicas, Faculdade de Medicina, Universidade de São Paulo	São Paulo (SP), Brazil
Sabrina dos Santos Pinheiro	Hospital de Clínicas de Porto Alegre	Porto Alegre (RS), Brazil
Sara Querido	Centro Hospitalar Lisboa Central, EPE	Lisbon, Portugal
Saulo Fernandes Saturnino	Hospital das Clínicas, Universidade Federal de Minas Gerais	Belo Horizonte (NG), Brazil
Sérgio Marba	Universidade Estadual de Campinas	Campinas (SP), Brazil
Sônia Testa Ramos	Instituto da Criança, Hospital das Clínicas, Faculdade de Medicina, Universidade de São Paulo	São Paulo (SP), Brazil
Soraia Forgiarini	Centro Universitário Metodista	Porto Alegre (S), Brazil
Taís S Rocha	Universidade Federal do Rio Grande do Sul	Porto Alegre (RS), Brazil
Tamer Hifnawy	Taibah University	Madinah, Saudi Arabia
Tatiana Barichello	University of Texas Health Science Center	Texas, United States
Tatiana Rech	Hospital de Clínicas de Porto Alegre	Porto Alegre (RS), Brazil
Teresa Cardoso	Centro Hospitalar do Porto	Porto, Portugal
Thiago Almeida	Universidade Federal de São Paulo	São Paulo (SP), Brazil
Thiago Correa	Hospital Israelita Albert Einste	São Paulo (SP), Brazil
Thiago Romano	Faculdade de Medicina do ABC	Santo André (SP), Brazil
Tiago Pereira	Hospital Curry Cabral	Lisbon, Portugal
Vandack Nobre	Universidade Federal de Minas Gerais	Belo Horizonte (MG), Brazil
Vanessa Lanziotti	Instituto de Puericultura e Pediatria Martagão Gesteira, Universidade Federal do Rio de Janeiro	Rio de Janeiro (RJ), Brazil
Vera Lúcia Krebs	Instituto da Criança, Hospital das Clínicas, Faculdade de Medicina, Universidade de São Paulo	São Paulo (SP), Brazil
Vicentes Souza-Dantas	Universidade Federal do Rio de Janeiro	Rio de Janeiro (RJ), Brazil
Viviane Bogado	Fundação Eletronuclear de Assistência Médica	Angra dos Reis (RJ), Brazil
Wagner Luis Nedel	Hospital Nossa Senhora da Conceição	Porto Alegre (RS), Brazil
William Manzanares	Faculdad de Medicina, Universidade de la Republica	Montevideo, Uruguay
Yeh-Li Ho	Hospital das Clínicas, Faculdade de Medicina, Universidade de São Paulo	São Paulo (SP), Brazil

